# Disentangling biases in responding from substantive effects in computational psychiatry

**DOI:** 10.1073/pnas.2624329123

**Published:** 2026-10-16

**Authors:** Claire M. Gillan, Stephen M. Fleming, Marion Rouault, Tricia X. F. Seow

**Affiliations:** ^a^ School of Psychology, Trinity College Dublin, Dublin D02 PN40, Ireland; ^b^ Trinity College Institute for Neuroscience, Trinity College Dublin, Dublin D02 PN40, Ireland; ^c^ FutureNeuro Research Ireland Centre, Trinity College Dublin, Dublin D02 PN40, Ireland; ^d^ Department of Experimental Psychology and Institute of Cognitive Neuroscience, University College London, London WC1H 0AP, United Kingdom; ^e^ Max Planck University College London Centre for Computational Psychiatry and Ageing Research, University College London, London WC1B 5EH, United Kingdom; ^f^ Sorbonne Université, Institut du Cerveau–Paris Brain Institute, Institut National de la Santé et de la Recherche Médicale, CNRS, Hôpital de la Pitié Salpêtrière, 47 boulevard de l’hôpital, Paris 75013, France; ^g^ Functional Imaging Laboratory, Department of Imaging Neuroscience, University College London, London WC1N 3AR, United Kingdom

Sarna, Dar, and Mazor (1) show how inattentive and biased (acquiescent) responding can inflate associations between mental health symptoms and metacognition, arguing this produces spurious confidence–compulsivity links in large online samples. We agree that considering these factors is important, and their paper advocates practical solutions for doing so. However, there are good reasons to doubt these factors fully account for the links between metacognition and mental health (2, 3).

No psychological measurement is free of noise or bias, and acquiescence is a substantive phenomenon that predates crowdsourcing by decades (4). Online platforms may exacerbate these issues—this is why convergence across samples is key. The confidence–compulsivity association appears not only in crowdsourced data but in samples with low inattention: patients recruited through closed channels (PIP study; 8.3% inattentive) (5) and unpaid citizen scientists (Neureka; <1% inattentive) (6). In these data, inattentive responding does not inflate compulsivity scores (Fig. 1 *A* and *B*) and both inattention and acquiescence are considerably smaller than in crowdsourced samples (1) (Fig. 1*C*).

**Fig. 1.**
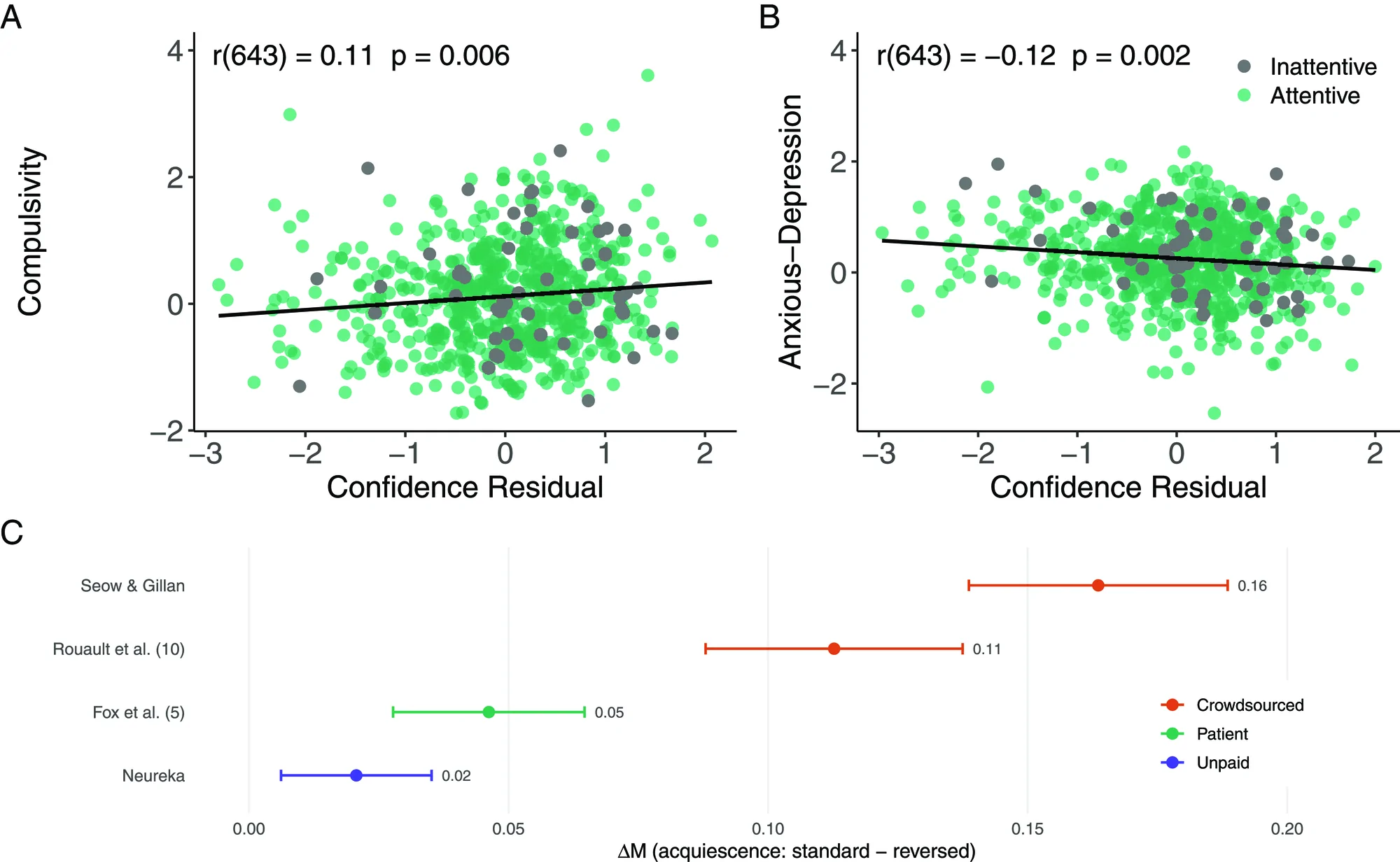
(*A* and *B*) Inattentive responding (in gray) has no impact on the associations between confidence and mental health dimensions in a large clinical sample (5). Confidence Residual refers to confidence scores residualized for age and gender and AD/CIT. (*C*) Markedly low levels of acquiescence-bias (defined as the difference between standard and reverse coded items in confidence correlation) were observed in both patient and unpaid participant data.

Sarna et al.’s work was motivated by apparently contradictory findings: Individuals with an obsessive-compulsive disorder (OCD) diagnosis display decreased confidence, while individuals high in compulsivity show increased confidence on similar tasks. However, compulsivity is not an OCD diagnosis but a transdiagnostic dimensional trait that also reflects aspects of schizotypy, alcohol use, impulsivity, and eating disorders (7). Sarna et al. do not measure compulsivity but speculate the issues “operate similarly, if not more strongly.” We think this conclusion premature; empirically, inattention actually affects compulsivity (d = 0.22) *less* than OCD symptoms (d = 0.31) (5), yet the confidence–compulsivity association is twice the size of that with OCD symptoms (8). As transdiagnostic, population-based research develops, we suggest the most critical issue to unpack is what it means to have a diagnosis versus scoring high on a trait (9).

Transdiagnostic methods emerged in part due to a recognition of the challenges that comorbidity presents for research in psychiatry. Anxious-depression and compulsivity are positively correlated (like many aspects of mental health), meaning specific or bidirectional effects may be obscured if they are not modelled jointly. The established approach therefore includes both dimensions as covariates in the same analysis to isolate their effects (5, 6, 8, 10). In a reanalysis of Sarna et al.’s own data using this model, we find that the confidence–compulsivity association persists after correcting for inattention and acquiescence (Table 1). Through a simulation, Sarna et al. show that controlling for depression can, under certain conditions, induce a spurious confidence–compulsivity effect even where none exists. However, this simulation does not adjudicate between causal structures; the observed data are equally compatible with a genuine effect of confidence on compulsivity that is obscured when depression is omitted. Distinguishing between these interpretations will require intervention studies or analyses that explicitly compare competing causal models.

**Table 1.** Associations between confidence and both OCD and depression under the standard covariate-adjusted model, with and without removal of inattentive responders and acquiescence correction

		Full sample (N = 188)		Attentive only (N = 142)		Attentive only + Acq. corr. (N = 142)	
		Est. (SE)	*P*-value	Est. (SE)	*P*-value	Est. (SE)	*P*-value
Model 1	Depression	−0.02 (0.01)	0.077	−0.02 (0.01)	0.075	−0.02 (0.01)	0.089
Model 2	OCD	0.05 (0.01)	<0.001***	0.03 (0.01)	0.069	0.02 (0.01)	0.193
Model 3	Depression	−0.04(0.01)	0.002**	−0.05 (0.02)	0.002**	−0.04 (0.02)	0.006**
	OCD	0.06 (0.01)	<0.001***	0.05 (0.02)	0.001**	0.04 (0.02)	0.009**
	Age	−0.02 (0.01)	0.195	−0.02 (0.01)	0.153	−0.02 (0.01)	0.126
	Sex	0.06 (0.02)	0.0097**	0.05 (0.03)	0.041*	0.06 (0.03)	0.042*

Models 1 and 2 are used by Sarna and colleagues (1). Model 3 includes covariates for co-occurring mental health symptoms and demographics (age and sex) variables. “Acq. Corr.” refers to the use of acquiescence-corrected mental health scores. That is, each item on the OCD and depression symptom scales are first residualized using a custom scale developed by Sarna et al. comprising “content-neutral” items, before being totaled for analysis. Two participants without sex data are removed.

We applaud Sarna et al. for their work, but there is a fine line between explaining variance in a phenomenon and explaining it away. Clinical constructs in psychiatry remain a work-in-progress and reconciling this debate will require further consideration to how these definitions impact our findings.

## Data Availability

Data and code data have been deposited in OSF (https://osf.io/df4wv). Previously published data were used for this work (5).
